# Neuropathological Diagnostic Considerations in Hyperkinetic Movement Disorders

**DOI:** 10.3389/fneur.2013.00007

**Published:** 2013-02-14

**Authors:** Wilfred F. A. den Dunnen

**Affiliations:** ^1^Department of Pathology and Medical Biology, University of Groningen, University Medical Center GroningenGroningen, Netherlands

**Keywords:** neuropathology, neurodegeneration, chorea, ballism, dystonia, tremor

## Abstract

Neuropathology of hyperkinetic movement disorders can be very challenging. This paper starts with basic functional anatomy of the basal ganglia in order to appreciate that focal lesions like for instance tumor or infarction can cause hyperkinetic movement disorders like (hemi)ballism. The neuropathology of different causes of chorea (amongst others Huntington’s disease, neuroacanthosis, and HLD-2) and dystonia (DYT1, PD, and Dopa-Responsive Dystonia) are described. Besides the functional anatomy of the basal ganglia a wider anatomical network view is provided. This forms the basis for the overview of the neuropathology of different forms of tremor.

## Introduction

Several clinical conditions are characterized by the presence of excessive involuntary movements and are grouped as hyperkinetic movement disorders. To these disorders belong chorea, ballism, dystonia, tremor, myoclonus, and tics. Many of these disorders can be explained by disturbances in the basal ganglia, but it is important to realize that disruptions outside the basal ganglia may also cause (combinations of) hyperkinetic movement disorders as well. A network view in which the basal ganglia participate may be necessary to understand the neurological symptomatology of a patient (Neychev et al., 2011). However, a basic understanding of the normal functional anatomy of the basal ganglia themselves can assist in the neuropathological evaluation of hyperkinesias.

There are many causes for disturbances in the basal ganglia and therefore only the most occurring will be described in more detail in this paper. It is important to understand, however, that some structural changes, due to for instance infarcts, hemorrhages, and tumors are not restricted to anatomical boundaries and thus may lead to complex (combinations of) movement disorders. Clinicopathological correlations can also be challenging with multiple affected areas lying in close proximity: choreatic or dystonic movement disorders may both be the result of damage of the striatum. Furthermore, the same type of movement disorder might be the result of damage at different anatomical locations. Last but not least it is important to understand that some movement disorders cannot be simply explained by changes at macro-anatomical level. These functional disturbances can for instance be caused by side effects of medication (Reich, 2010).

Clinicopathological correlation is an established method to link neurological (dys)function to a specific/responsible brain region. In that regard the neuropathologist should work together with the patient’s clinicians. There are, however drawbacks to this method (Neychev et al., 2011). Routine histology may not show any tissue changes, but application of more sophisticated methods may be necessary, for instance using specific monoclonal antibodies. Questions may remain whether a certain type of histopathological lesion is also responsible for the neurological disorder. Furthermore, as mentioned earlier, evaluating histopathology could be biased toward structural changes/defects, whereas metabolic or physiological disturbances may be more relevant.

In the following, first the functional neuroanatomy of the basal ganglia in health and disease will be described. Thereafter, chorea, ballism, dystonia, and tremor will be described with their differential diagnoses and the (specific) neuropathological changes when present. As we shall see, for some forms of hyperkinetic movement disorders there is surprisingly little neuropathological data available in the literature.

## Functional Neuroanatomy and the Relation with Movement Disorders

The basal ganglia encompass the striatum (caudate nucleus and putamen), globus pallidus, subthalamic nucleus (STN), and substantia nigra. This “extrapyramidal” system constitutes a number of interrelated circuits from which output emerges at different levels. Although the cortical pyramidal and subcortical extrapyramidal systems do not work independently from one another, the term extrapyramidal motor system is convenient, because its centers are (at least to some extent) implicated in the pathogenesis of several movement disorders including the hyperkinesias.

Besides motor control, which is mostly originating from the lateral compartments of the basal ganglia, there also exist “ventral” and “limbic” compartments, involved in cognitive functions such as learning and memory. The nucleus accumbens and substantia innominata are considered the ventral portions of the striatum and globus pallidus respectively. Also the accumbens receives dopaminergic input originating from the ventral tegmental area. I mention this specifically, because basal ganglia pathology may also lead to non-motor changes/pathology. Lesions of the caudate nucleus, for instance, may lead to apathy with loss of initiative in a significant portion of the patients (Bhatia and Marsden, 1994).

### Input

**Figure 1:**
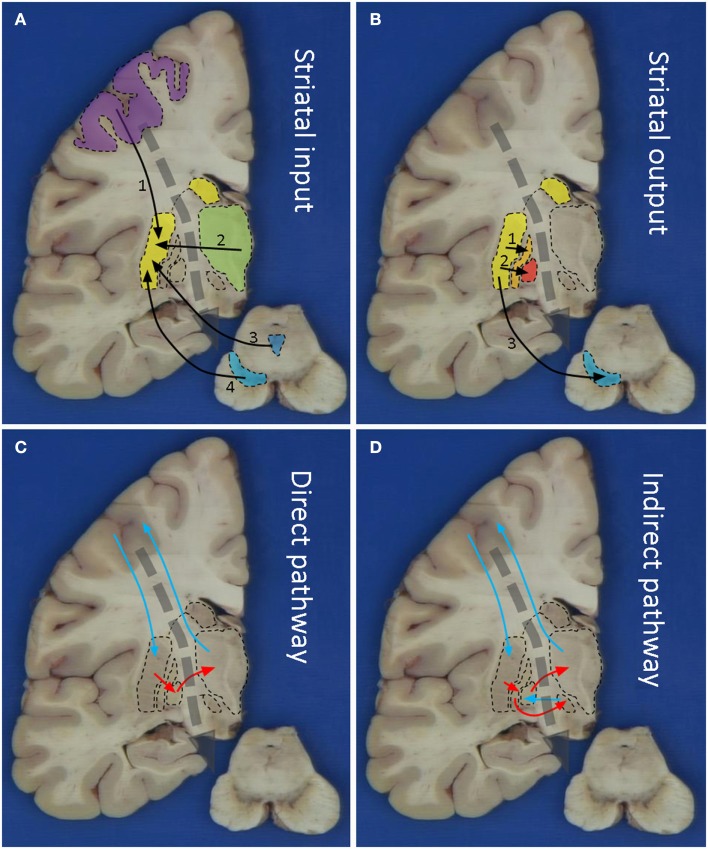
**Macroscopic pictures of human brain sections with superimposed schematic drawings showing striatal input (A), striatal output (B), the direct (C), and indirect (D) pathways**. Striatal input comes from 1: motor cortex, 2: thalamus, 3: oral raphe nuclei, and 4: substantia nigra. Striatal output is via 1: extern pallidum, 2: intern pallidum, and 3: substantia nigra. The blue arrows in **(C)** and **(D)** indicate stimulatory/glutamatergic neurotransmission and the red arrows inhibitory/GABAergic neurotransmission.

The striatum is the most important input system of the basal ganglia receiving glutamatergic fibers from the cerebral cortex, in particular the motor areas of the frontal lobe (Brodmann areas 4 and 6; Figure 1A). These fibers are topically organized and originate from projection neurons of the fifth layer (Alexander et al., 1986). Besides the motor and premotor cortices, further inputs come from the centromedian nucleus of the thalamus, substantia nigra, and oral raphe nuclei (Figure 1A).

The striatum consists of two compartments: the patch and the extrastriosomal matrix. These receive different inputs and have different targets. Imbalanced activity between these compartments likely underlies the occurrence of stereotypies (Saka et al., 2004). Specific degeneration of the striatum occurs in Huntington’s disease (HD), a well known cause for chorea. Also in other forms of neurodegenerative diseases, the striatum may be affected, for instance in Multiple System Atrophy (MSA) and Cortico-Basal Degeneration (CBD). Damage to the striatum may also be the result from toxicity, for instance in Wilson’s disease. It is, however, important to realize that these diseases might also affect other brain areas, thus resulting in a more complex clinical picture.

Besides the neocortex, glutamatergic innervation of the striatum also comes from the thalamus; especially the caudal intralaminar nuclei. Deep Brain Stimulation (DBS) of these nuclei can alleviate some of the symptoms seen in Parkinson’s disease (PD) and Tourette’s syndrome. Interestingly, thalamic inputs generate more profuse fields of axon terminals than do cortical axons. Furthermore, thalamic fibers may innervate some striatal interneurons, but not other populations of striatal neurons. The variegated glutamatergic innervations by neocortex and thalamus gives rise to a complex picture of striatal stimulation and damage to either of these input systems could cause a neurochemical disbalance in the striatum. For instance, post-mortem studies in PSP, HD, or PD patients showed up to 50% neuronal loss in the centromedian thalamic complex, thereby contributing to the clinical picture (Henderson et al., 2000).

Apart from glutamatergic innervation, the striatum receives input from dopaminergic midbrain neurons, especially from the ventral part of the Substantia Nigra pars compacta (SNc), which is the most sensitive region in PD (Kish et al., 1998). The loss of dopaminergic mediated regulation of striatal transmission is typical in PD. Furthermore, in PD massive loss of spines in the striatum likely contributes to imbalances in processing extrinsic inputs to the striatum. On the contrary, heightened dopaminergic activity may induce chorea, as can be seen in levodopa treated PD patients or in patients who develop dopamine supersensitivity from chronic neuroleptic therapy (tardive dyskinesia; Shoulson, 1986). Also in a rat HD model a hyperdopaminergic status was found that was not necessarily related to striatal shrinkage (Jahanshahi et al., 2010).

Additionally, the striatum receives serotonergic input from the oral raphe nuclei and noradrenergic innervations from the locus coeruleus. Both of these nuclei show degenerative changes in a variety of neurodegenerative diseases (amongst others PD) and contribute to the clinical picture (Braak et al., 2003; Alafuzoff et al., 2009).

### Output

**Figure 2:**
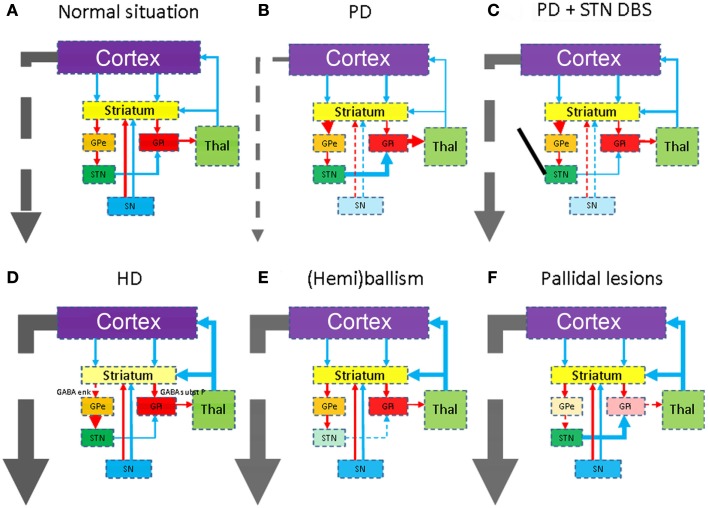
**Schematic representations of the combined direct and indirect pathways in health (A) and disease**. The colors of the anatomical structures and the arrows correspond with Figure 1. As Parkinson’s disease is the second most occurring form of neurodegenerative disease, it is mentioned here in **(B)**. Due to degeneration of the substantia nigra (SN), the subthalamic nucleus (STN) becomes hyperactive. STN deep brain stimulation **(C)** restores this disbalance again. Degeneration of the striatum in for instance Huntington’s disease **(D)**, the STN in for instance infarcts **(E)**, or the pallidum **(F)** will lead to different forms of hyperkinesia.

The output of the striatum is organized via the external and internal segments of the Globus Pallidus (GPe/GPi), thus giving rise to the indirect and direct pathways respectively (Figures 1B–D). The concept of direct and indirect pathways in the basal ganglia can help to explain some of the movement disorders described in this article (see also Figure 2). Furthermore this concept lead to the development of surgical approaches aimed at for instance the GPi (Obeso et al., 2008).

The Subthalamic Nucleus is a small nucleus in the indirect pathway and considered the pacemaker of the basal ganglia (Smith, 2012). The STN is the primary surgical target in PD disease (see Figure 2C). Specific degeneration of the STN is considered the cause of ballism (see Figure 2E). However, defective disinhibition of the thalamus by the STN may also produce chorea (Johnson and Fahn, 1977). Often the onset is sudden and caused by a small infarction or hemorrhage. It is however important to realize that also the STN is more or less affected in various forms of neurodegenerative diseases.

This global overview of the functional anatomy of the basal ganglia may help in the neuropathological evaluation (for instance in the search of focal lesions) and correlation with the clinical picture. The following part of this article will describe the differential diagnosis of the different hyperkinesias and the typical neuropathological pictures of the more occurring diseases.

## Neuropathology of Chorea

There are many causes of chorea, which may be hereditary, secondary (infections, immunologic, drug-induced, metabolic, endocrine, or vascular), or of unknown etiology (Shoulson, 1986; Mark, 2012). Especially some of the most occurring hereditary neurodegenerative diseases with rather specific histopathological reaction patterns will be described here.

**Figure 3:**
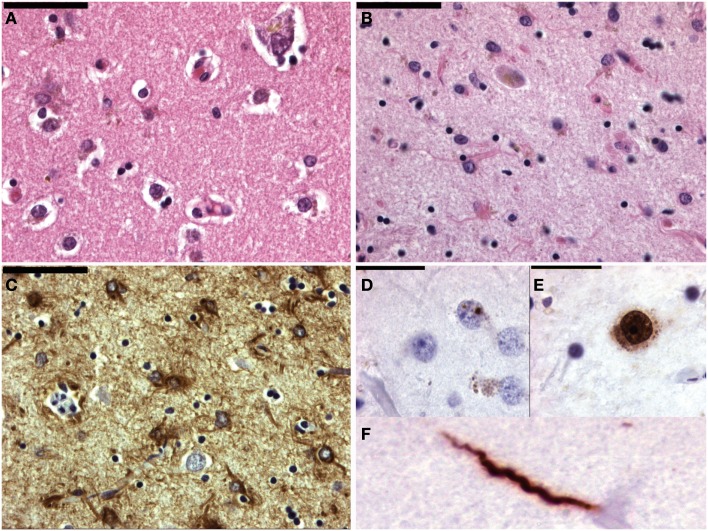
**Micrographs of the striatum in Huntington’s disease**. **(A)** Shows a normal age matched control (H&E staining). In **(B)** the number of neurons is lower and astrogliosis is already apparent. **(C)** Shows a GFAP immunohistochemical staining highlighting the astrogliosis. In **(D)** the hallmark lesion: neuronal intranuclear inclusion can be observed. **(E**,**F)** Show 1c2 immunohistochemistry. In neuron in **(E)** also shows diffuse nuclear staining as well as granular cytoplasmic staining. **(F)** Shows aggregated polyQ protein in a neurite.

Huntington’s disease usually appears in adult life and is caused by a mutation in the huntingtin (htt) gene. This progressive neurodegenerative disease is characterized by a movement disorder, dementia, and personality changes. Typically the striatum shows various degrees of degeneration/neuronal loss (Vonsattel et al., 1985) and gliosis (Figure 3). Widespread cortical atrophy may also be present. Less well known, is probably the degeneration of the premotor oculomotor brainstem system and the cerebellum (Rub et al., 2009, 2012).

In the mutated htt-gene an elongated CAG-repeat is present, giving rise to an elongated polyglutamine (polyQ) stretch, which accumulates during breakdown, in turn giving rise to proteinaceous inclusions. Using htt-specific antibodies, or more general polyQ-antibodies like the 1C2-clone, various forms of protein aggregations can be observed (Figure 3). Besides the hallmark lesion, e.g., the neuronal intranuclear inclusion (NII), diffuse nuclear staining, granular cytoplasmic staining, and neuritic staining may be observed (Figure 3). Other polyQ-diseases [like several of the spino-cerebellar ataxias (SCA), dentato-rubro-pallido-luysian atrophy (DRPLA), or HD-Like 2 (HDL2)] show similar staining patterns (Seidel et al., 2010, 2011). They can be differentiated however, based upon the variegated neuroanatomical degeneration throughout the nervous system and the density/neuroanatomical distribution of the different inclusion patterns (Seidel et al., 2012).

Other conditions in which chorea is a major manifestation can often be excluded on clinical grounds. The most common other form of hereditary adult-onset chorea is neuroacanthosis, presenting with mild chorea, tics, feeding dystonia, and red cell acanthocytes. Interestingly as with HD, a young age at onset is more likely to produce parkinsonism and dystonia rather than chorea. Neuropathology is similar to HD with regard to the striatal neurodegeneration and gliosis. PolyQ-inclusion bodies, however, are absent.

Dentato-Rubro-Pallido-Luysian Atrophy is another autosomal disorder, mainly described in Japanese and the result of an expanded CAG-repeat (Seidel et al., 2012). DRPLA clinically overlaps with HD and shows combinations of chorea, myoclonus, seizures, ataxia, and dementia. Neurodegeneration is centered in the cerebello and pallidofugal systems, but degenerative changes as well as polyQ inclusions can be found in many nuclei.

Other PolyQ-disorders that can present with chorea are SCA17 and HDL2. Whereas the types of protein inclusions may be the same as in HD, the density and distribution patterns differ amongst the various diseases.

HDL1 is caused by an octapeptide repeat in the prion protein and HLD3 is a misnomer as it appears to be an autosomal-recessive neurodegenerative disorder beginning in early childhood and besides chorea and dystonia characterized by spasticity, seizures, mutism, intellectual impairment, and neuropathologically with frontal and striatal degeneration.

Senile chorea is a late-onset generalized chorea with no family history or dementia and should be considered a diagnose per exclusionem. Slow progression of the intensity and extent of the involuntary movements occurs. With DNA testing, up to 50% of these patients do show a CAG-expansion in the htt-gene. This could be due to (1) a *de novo*-mutation or (2) the fact that the patient is ignorant of the family history or (3) may deny that history. Neuropathological evaluation of the striatum therefore should include anti-PolyQ immunohistochemistry or more general inclusion markers like ubiquitin and/or p62 (Seidel et al., 2009). When positive a more extensive range of anatomical structures should be evaluated with the same antibodies.

Still other patients with late-onset generalized choreatic movement disorders can be diagnosed with for instance Fahr’s disease, or antiphospholipid antibody syndrome. Despite extensive clinicopathological evaluation a patient can remain undiagnosed and go under the term senile chorea. Degenerative and gliotic changes are found in the caudate nucleus and putamen, but not to the extent as seen in HD.

Unilateral and mostly more violent choreatic movements may develop in middle-aged or elderly patients. The sudden onset suggests a (strategic) vascular basis, which can both be hemorrhagic or ischemic. Much less common are tumors or plaques in Multiple Sclerosis. Obviously the neuropathological reaction patterns in these diseases are not specific for the basal ganglia.

## Neuropathology of Dystonia

**Figure 4:**
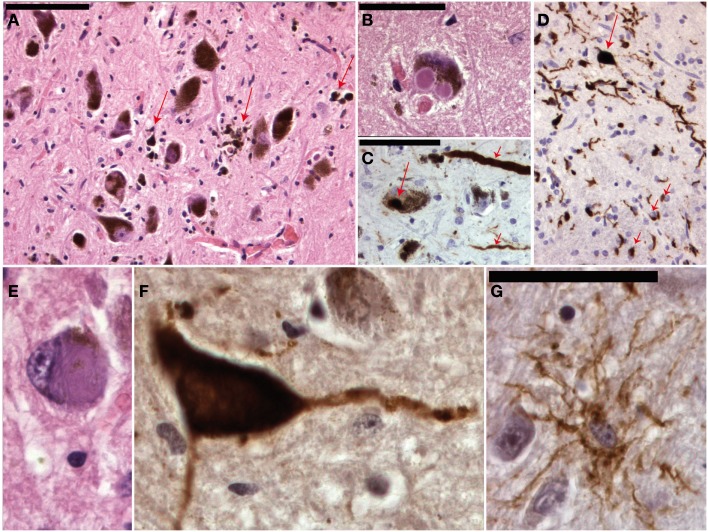
**Micrographs of the mesencephalon in Parkinson’s disease (A–C), mutiple system atrophy (D) and progressive supranuclear palsy (E–G)**. **(A)** Shows an H&E staining of the substantia nigra with neuronal degeneration and phagocytosis of neuromelanin (arrows). **(B)** Shows a neuron with two classical Lewy bodies. **(C)** Shows alfa-synuclein staining with positive staining of a Lewy body (long arrow) and Lewy neuritis (short arrows). MSA is also an alfa-synucleinopathy **(D)** with both neuronal (long arrow) and oligodendroglial (short arrows) inclusions. **(E)** Shows a neuron in PSP with a filamentous basophilic inclusion (H&E staining). **(F,G)** Show immunohistochemical stainings for hyperphosphorylated tau-protein (AT8 clone) with inclusions in a neuron and “tufted” astrocyte respectively.

The term dystonia was coined by Oppenheim in 1911 and is nowadays defined as a syndrome of sustained muscle contraction, frequently causing twisting and repetitive movements or abnormal postures (Fahn, 1984). Dystonia can be classified by age of onset, by distribution (focal, segmental, generalized, or hemidystonia) or by etiology (primary/idiopathic, secondary, or heredodegenerative). Gross and light microscopical examination does not reveal consistent histomorphological changes. In fact there is surprisingly little data on the histopathological features of dystonia (Standaert, 2011). Striatal and dopaminergic dysfunction is suspected to play a role, which is based upon: (1) imaging or pathology of focal (unilateral) lesions in the putamen, leading to (hemi)dystonia, (2) pathology that can be seen in non-primary dystonias, such as infarction of the lentiform nucleus, Wilson disease, glutaric aciduria, or neurodegeneration with brain iron accumulation, (3) combinations with other movement disorders with known involvement of the basal ganglia such as PD (Figure 4) or HD, (4) neurosurgical interventions in the basal ganglia that can alleviate dystonia, (5) sophisticated imaging methods showing abnormalities in the activity of the basal ganglia, and (6) animal studies (Neychev et al., 2011). It is, however, important to mention that there is accumulating evidence that abnormalities outside the basal ganglia (cerebral cortex, cerebellum, brainstem nuclei) can also cause dystonia (Argyelan et al., 2009).

The number of autopsy reports on primary dystonia is very limited and the first gene (DYT1) was not identified until 1997. Reports before this date thus lack the appropriate genetic background. The articles before 1997 describe various results: some patients showed neurodegeneration in the SNc, Locus Coeruleus, raphe nuclei, Pedunculo-Pontine Nucleus (PPN), and dentate ncl, while some patients did not show any histopathological changes at all (Kulisevsky et al., 1988; Zweig et al., 1988; Mark et al., 1994). Furthermore, in some patients with neurodegenerative changes neurofibrillary tangles (Zweig et al., 1988) or Lewy body pathology (Kulisevsky et al., 1988; Mark et al., 1994) could be found and it is therefore questionable whether some of these cases were indeed primary (pure) forms of dystonia and not forms of CBD/PSP (Figure 4) or Diffuse Lewy Body Disease.

Also from genetically confirmed cases of dystonia little neuropathological data is available. In four papers a total of 10 DYT1-autopsies were described, which showed no signs of neuronal loss, inflammation, or lack of dopamine content (Furukawa et al., 2000; Augood et al., 2002; Walker et al., 2002). McNaught et al. (2004) described four additional DYT1 cases and found ubiquitin and torsinA-positive (Rostasy et al., 2003) neuronal inclusions in several brainstem nuclei. Although these observations have not been replicated by others, similar inclusions have been found in DYT1 mouse models (Granata et al., 2009). No pathology has been described so far for DYT6 and DYT11.

Dopa-responsive dystonia (DRD) is, as the name implies, a group of disorders that show clinical improvement with dopaminergic therapies. DYT5 (also called Segawa disease) is the best described form. DYT14 is related to DYT5 and autopsy studies in these patients show marked depigmentation of neurons in de SN. In one case Lewy bodies were described in the SN, but in other cases no neurodegeneration or inclusions were observed.

Dystonia is a feature that can occur in a wide range of destructive or neurodegenerative diseases. Perhaps the most common degenerative cause of dystonia is PD! Other degenerative diseases that might cause dystonia-plus syndromes are PSP, CBD, and MSA (Figure 4). Hereditary forms may be autosomal dominant (for instance HD, SCA3, Fahr disease, DRPLA, etc), autosomal-recessive [for instance Gangliosidosis, Ataxia Telangiectasia, Ataxia with Vitamin E Deficiency (AVED)], X-linked (for instance Lubag/DYT3), or mitochondrial (for instance Leigh, MERFF/MELAS; Fahn et al., 1998).

Apart from basal ganglia pathology, also changes in the brainstem and cerebellum may induce dystonic symptoms (Argyelan et al., 2009). Interestingly, cervical dystonia has been described to be triggered by posterior fossa tumors. Furthermore, also in familial forms of ataxia, dystonic features have been described. As we shall see in the next part of this article other forms of hyperkinetic movement disorders like tremors may also occur due to lesions outside the basal ganglia circuitry.

## Neuropathology of Tremor

**Figure 5:**
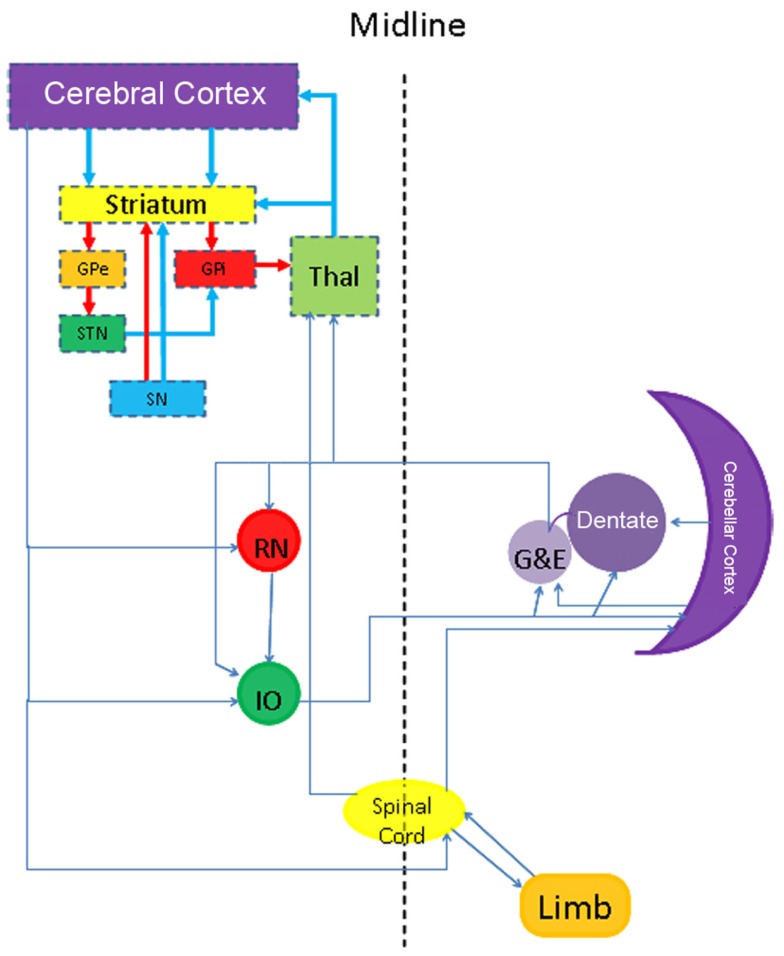
**Scheme showing a more extensive neuroanatomical view to understand some forms of dystonia and tremor**.

Many forms of tremor exist and distinctions can be made based upon clinical grounds and electrophysiological recordings. Besides which body parts shows tremor, a distinction can be made whether the tremor occurs in rest, during movement or intentional. Before discussing the more common forms of tremor and the rather limited (specific) neuropathology, it is necessary to broaden our view of the functional neuroanatomy. Based upon focal lesions as well as advanced imaging it has become clear that the cerebellar cortex, some deep cerebellar nuclei (dentate, globose, and emboliform), red nucleus, inferior olive, spinal cord (tracts), and peripheral nerves have to be added to the circuitry described earlier (Figure 5). PET studies have shown increases in blood flow in the cerebellar cortex in many forms of tremor, such as tremor in PD and Essential tremor (ET; Boecker and Brooks, 1998). The cerebellum receives input from many sources and projects to the thalamus, red nucleus and inferior olive. Whether the cerebellar hyperactivity is cause or a non-specific consequence of the tremor is matter of debate. The same can be said from the nucleus ventralis intermedius of the thalamus, even though it is the preferred location for DBS in virtually every form of tremor (Schuurman et al., 2000; Krauss et al., 2001). It could well be that the Vim and its connections facilitate the development of the tremor, but that the source can originate from virtually any location in the motor system shown in Figure 5 (Elble, 2012).

**Figure 6:**
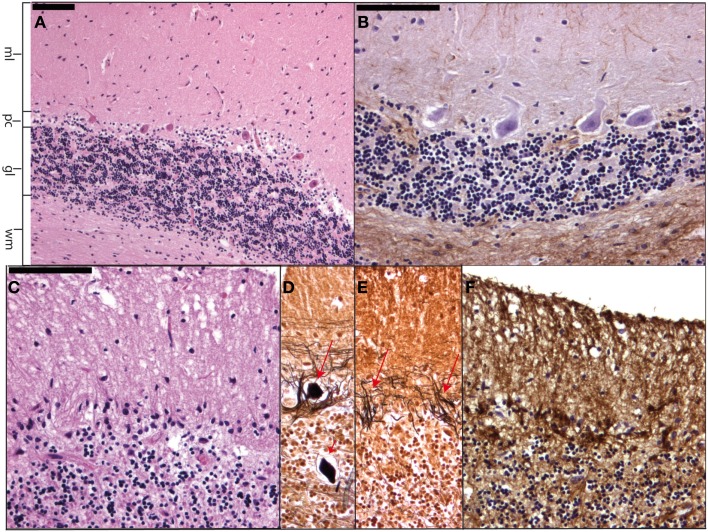
**Micrographs showing a normal cerebellar cortex (A) and (B) as well as cerebellar cortical degeneration (C–F)**. In **(A)** (H&E staining) and **(B)** (GFAP staining) the molecular layer (ml), Purkinje cell layer (pc), granular layer (gl), and cerebellar white matter (wm) can be readily distinguished. **(C–F)** Show respectively Purkinje cell and granular cell loss with formation of axonal torpedoes [short arrow in **(D)]**, empty baskets [arrows in **(E)]**, and Bergmann astrogliosis where the Purkinje cells have disappeared.

Essential tremor is the most occurring form of tremor and can occur at any age, although the prevalence increases with age. The hallmark of this progressive disease is a kinetic tremor of the arms although other types of tremors and other motor (ataxia) and non-motor (dementia) symptoms may occur (Benito-León, 2008). Although many patients have a positive family history, genetic linkage studies have not found a gene yet. In the relatively scarce post-mortem examinations of ET brains degenerative changes have been seen in the cerebellar cortex with loss of Purkinje cells (PC) and the formation of PC axonal torpedoes (Figure 6). In a smaller number of brains also Lewy bodies have been found in the brain stem with relatively normal cerebella (Louis et al., 2007; Louis, 2010). Furthermore, one other case of ET had ubiquitin and p62 positive PC intranuclear inclusions. In this case no pathological tau-protein, TDP43 or alfa-synuclein was found. Genetic analysis excluded SCA1, SCA2, SCA3, SCA6, SCA7, and Fragile X Tremor Ataxia Syndrome (Louis et al., 2012).

In PD a rest tremor is mostly observed, but action tremors also frequently occur. In animal studies destruction of the SNc with MTPT lead to both forms of tremors (Bergman et al., 1998). The hallmark of PD is neuronal loss of the SNc. Many parts of the motor system seem to be involved in the production of the rest tremor as DBS of STN, pallidum, and ventrolateral thalamus can all suppress the tremor.

The cerebellar intention tremor is often irregular and affects the proximal limb muscles more than the distal ones. Typically patients present with lesions in the cerebellum (Figure 6) or the fiber tracts from the deep cerebellar nuclei (dentate, globose, emboliform) to the contralateral ventrolateral thalamus. Small lesions in the vicinity of the ventrolateral thalamus may cause an intention tremor, but without ataxia (Qureshi et al., 1996).

Holmes tremor shows a combination of resting, postural and kinetic tremor. Lesions in the vicinity of the ventrolateral thalamus and/or the red nucleus as well as surrounding fiber tracts may explain this combination of tremors. Holmes tremor typically begins weeks to months after a thalamic or midbrain stroke. Therefore, secondary or compensatory mechanisms in the motor system, rather than the damaged compartments themselves may cause this tremor. Post-stroke tremor without lesions in the midbrain, superior cerebellar peduncle, or the cerebellum is very rare.

Palatal tremor (also called palatal myoclonus) occurs in two forms: symptomatic and essential. In the former (which often shows other brainstem and cerebellar signs) neuropathology has found secondary hypertrophy of the inferior olive as a consequence of damage to the dentate-olivary pathway. In the latter an annoying ear clicking is present without other neurological symptoms and the pathology is not clear.

It is important to realize that many other tremors exist, such as orthostatic tremor, tremor due to peripheral nerve pathology, drug-induced tremor, and psychogenic tremor. For neuropathology it is interesting to mention persistent lithium-induced tremor accompanied by ataxia, as patients may show neuronal loss and gliosis of the cerebellar cortex (Figure 6) and dentate nuclei (Schneider and Mirra, 1994).

## Concluding Remarks

For neuropathological diagnostics of hyperkinetic movement disorders it is not only necessary to have a good understanding of the functional anatomy of the basal ganglia, but also of a wider network view that includes other structures (amongst others the cerebellum). Besides focal lesions, samples should also be taken from normal appearing brain areas in the above mentioned structures in order to look for specific reaction patterns/neurodegeneration. A series of additional stains to evaluate these patterns more clearly will be necessary as well as ubiquitin or p62 stainings to evaluate the general presence of proteinaceous aggregated material. Depending on the type and distribution of the aggregates more specific immunohistochemical stains can be added (see also Table 1). Contact with the referring clinician is warranted in order to correlate the neuropathological findings with the clinical picture. The neuropathologist should keep in mind that direct clinicopathological correlations are not always present and that functional disturbances could also cause hyperkinesias.

**Table 1:** **known neuropathology in hyperkinetic disorders described in this article**.

Hyperkinetic disorder	Disease	Description of neuropathology	Immunohistochemistry	Future directions
Chorea	HD	Degeneration in striatum, frontal cortex, brain stem, cerebellum + gliosis + protein inclusions	1C2+/huntingtin+	
	Neuroacanthosis	Striatal degeneration + gliosis (no protein inclusions)	1C2−	
	DRPLA	Degeneration in pallidum, STN, dentate, and red nucleus + protein inclusions	1C2+/atrophin-1	
	SCA17	Degeneration in striatum, ventral thalamus, SN, inferior olive, cerebellum + protein inclusions	1C2+/TBP	
	HDL2	Degeneration and protein inclusions largely comparable to HD	1C2+	
	HDL1	??		Neuropathology?
	HDL3	??		Neuropathology?
Dystonia	DYT1	Degeneration of SNc, LC, raphe nuclei, PPN, and dentate	Ub+, TorsinA+	Concomitant tangles/Lewy bodies?
	DYT6 and DYT11	??		Neuropathology?
	DYT5/DYT14	SN neurodegeneration; 1 case with Lewy bodies		Concomitant Lewy bodies?
	LUBAG/DYT3	Mosaic neuronal loss in caudate and lateral putamen + gliosis		
	PD	Diffuse degeneration throughout the CNS (six stages) with Lewy bodies, pale bodies, and Lewy neurites	Alfa-synuclein	
	PSP	Degeneration of SN, locus coeruleus, pons, midbrain, pallidum + neuronal and glial protein inclusions	tau	
	CBD	Degeneration in SN, pallidum, striatum, STN, thalamus, and red nucleus + neuronal and glial protein inclusions	tau	
	MSA	Variable degeneration of olivo-ponto-cerebellar system and basal ganglia + neuronal + glial protein inclusions	Alfa-synuclein
Tremor	ET	Cerebellar cortical degeneration		LB in brainstem concomitant? Ub+/p62+ nii specific?
	PD	See above	Alfa-synuclein	
	Cerebellar tremor	Lesions in cerebellum or fiber tracts from deep cerebellar nuclei		
	Holmes tremor	Lesions in ventrolateral thalamus/red nucleus or surrounding fiber tracts		
	Palatal tremor	Degeneration of dentate-olivary pathway + secondary hypertrophy of inferior olive		
	Li induced tremor	Neuronal loss + gliosis in cerebellar cortex and dentate nucleus		

## Conflict of Interest Statement

The author declares that the research was conducted in the absence of any commercial or financial relationships that could be construed as a potential conflict of interest.

## Abbreviations

AVED, ataxia with vitamin E deficiency; CBD, cortico-basal degeneration; CM/PF, centromedian nucleus; DBS, deep brain stimulation; DRD, dopa-responsive dystonia; DRPLA, dentato-rubro-pallido-luysian atrophy; ET, essential tremor; GPi, globus pallidus internal segment; HD, Huntington’s disease; HDL-1, -2, -3, Huntington’s disease-like-1, -2, -3; MELAS, mitochondrial encephalomyopathy, lactic acidosis, and stroke-like episodes; MERFF, myoclonic epilepsy with ragged red fibers; MS, multiple sclerosis; MSA, multiple system atrophy; MTPT, 1-methyl-4-phenyl-1,2,3,6-tetrahydropyridine; nii, neuronal intranuclear inclusion; PD, Parkinson’s disease; PET, positron emission tomography; PPN, pedunculo-pontine nucleus; SCA, spino-cerebellar ataxia; SNc, substantia nigra pars compacta; STN, subthalamic nucleus; TDP43, TarDNA-binding protein 43.

## References

[B1] Alafuzoff I. Ince P. G. Arzberger T. Al-Sarray S. Bell J. Bodi I. (2009). Staging/typing of Lewy body related alpha-synuclein pathology: a study of the BrainNet Europe Consortium. Acta Neuropathol. 117, 635–652 10.1007/s00401-009-0485-4 19330340

[B2] Alexander G. E. DeLong M. R. Strick P. L. (1986). Parallel organization of functionally segregated circuits linking basal ganglia and cortex. Annu. Rev. Neurosci. 9, 357–381 10.1146/annurev.ne.09.030186.002041 3085570

[B3] Argyelan M. Carbon M. Niethammer M. Ulug A. M. Voss H. U. Bressman S. B. (2009). Cerebellothalamocortical connectivity regulates penetrance in dystonia. J. Neurosci. 29, 9740–9747 10.1523/JNEUROSCI.2300-09.2009 19657027 PMC2745646

[B4] Augood S. J. Hollingsworth Z. Albers D. S. Yang L. Leung J. C. Muller B. (2002). Dopamine transmission in DYT1 dystonia: a biochemical and autoradiographical study. Neurology 59, 445–448 10.1212/WNL.59.3.445 12177384

[B5] Benito-León J. (2008). Essential tremor: from a monosymptomatic disorder to a more complex entity. Neuroepidemiology 31, 191–192 10.1159/000154933 18784419

[B6] Bergman H. Raz A. Feingold A. Nini A. Nelken I. Hansel D. (1998). Physiology of MPTP tremor. Mov. Disord. 13, 29–34 10.1002/mds.870130109 9827591

[B7] Bhatia K. P. Marsden C. D. (1994). The behavioural and motor consequences of focal lesions of the basal ganglia in man. Brain 117, 859–876 10.1093/brain/117.4.859 7922471

[B8] Boecker H. Brooks D. J. (1998). Functional imaging of tremor. Mov. Disord. 13(Suppl. 3), 64–72 10.1002/mds.870131311 9827597

[B9] Braak H. Del Tredici K. Rub U. de Vos R. A. I. Jansen Steur E. N. H. Braak E. (2003). Staging of brain pathology related to sporadic Parkinson’s disease. Neurobiol. Aging 24, 197–211 10.1016/S0197-4580(02)00065-9 12498954

[B10] Elble R. J. (2012). “Pathophysiology of tremor” in Movement disorders, 3rd Edn, Chap. 27, eds Watts R. L. Standaert D. Obeso J. A. (McGraw Hill), 539–554

[B11] Fahn S. (1984). The varied clinical expressions of dystonia. Neurol. Clin. 2, 541–554 6398404

[B12] Fahn S. Bressman S. B. Marsden C. D. (1998). Classification of dystonia. Adv. Neurol. 78, 1–10 9750897

[B13] Furukawa Y. Hornykiewicz O. Fahn S. Kish S. J. (2000). Striatal dopamine in early-onset primary torion dystonia with the DYT1 mutation. Neurology 54, 1193–1195 10.1212/WNL.54.11.2187 10720299

[B14] Granata A. Schiavo G. Warner T. T. (2009). TorsinA and dystonia: from nuclear envelope to synapse. J. Neurochem. 109, 1596–1609 10.1111/j.1471-4159.2009.06095.x 19457118

[B15] Henderson J. M. Carpenter K. Cartwright H. Halliday G. M. (2000). Degeneration of the centre median-parafascicular complex in Parkinson’s disease. Ann. Neurol. 47, 345–352 10.1002/1531-8249(200003)47:3<345::AID-ANA10>3.0.CO;2-V 10716254

[B16] Jahanshahi A. Vlamings R. Kaya A. H. Lim L. W. Tan S. Visser-Vandewalle V. (2010). Hyperdopaminergic status in experimental Huntington disease. J. Neuropathol. Exp. Neurol. 69, 910–917 10.1097/NEN.0b013e3181ee005d 20720506

[B17] Johnson W. G. Fahn S. (1977). Treatment of vascular hemiballism and hemichorea. Neurology 27, 634–636 10.1212/WNL.27.7.634 559968

[B18] Kish S. J. Shannak K. Hornykiewicz O. (1998). Uneven pattern of dopamine loss in the striatum of patients with idiopathic Parkinson’s disease. Pathophysiologic and clinical implications. N. Engl. J. Med. 318, 876–880 10.1056/NEJM198804073181402 3352672

[B19] Krauss J. K. Simpson R. K. Jr. Ondo W. G. Pohle T. Burgunder J. M. Jankovic J. (2001). Concepts and methods in chronic thalamic stimulation for treatment of tremor: technique and application. Neurosurgery 48, 535–543 10.1097/00006123-200103000-00015 11270543

[B20] Kulisevsky J. Marti M. J. Ferrer I. Tolosa E. (1988). Meige syndrome: neuropathology of a case. Mov. Disord. 3, 170–175 10.1002/mds.870030209 3221903

[B21] Louis E. D. (2010). Essential tremor: evolving clinicopathological concepts in an era of intensive post-mortem enquiry. Lancet Neurol. 9, 613–622 10.1016/S1474-4422(10)70090-9 20451458

[B22] Louis E. D. Faust P. L. Vonsattel J. P. Honig L. S. Rajput A. Robinson C. A. (2007). Neuropathological changes in essential tremor: 33 cases compared with 21 controls. Brain 130, 3297–3307 10.1093/brain/awm266 18025031

[B23] Louis E. D. Mazzoni P. Ma K. J. Moskowitz C. B. Lawton A. Garber A. (2012). Essential tremor with ubiquitinated intranuclear inclusions and cerebellar degeneration. Clin. Neuropathol. 31, 119–126 22551915 10.5414/NP300414 PMC3636555

[B24] Mark M. H. (2012). “Other choreatic disorders” in Movement Disorders, 3rd Edn, Chap. 36, eds Watts R. L. Standaert D. Obeso J. A. (McGraw Hill), 725–748

[B25] Mark M. H. Sage J. I. Dickson D. W. Herikkila R. E. Manzino L. Schwarz K. O. (1994). Meige syndrome in the spectrum of Lewy body disease. Neurology 44, 1432–1436 10.1212/WNL.44.8.1432 8058144

[B26] McNaught K. S. Kapustin A. Jackson T. Jengelley T. A. Jnobaptiste R. Shashidharan P. (2004). Brainstem pathology in DYT1 primary torsion dystonia. Ann. Neurol. 56, 540–547 10.1002/ana.20186 15455404

[B27] Neychev V. K. Gross R. E. Lehericy S. Hess E. J. Jinnah H. A. (2011). The functional neuroanatomy of dystonia. Neurobiol. Dis. 42, 185–201 10.1016/j.nbd.2011.01.026 21303695 PMC3478782

[B28] Obeso J. A. Rodriguez-Oroz M. C. Benitez-Temino B. Blesa F. J. Guridi J. Marin C. (2008). Functional organization of the basal ganglia: therapeutic implications for Parkinson’s disease. Mov. Disord. 23(Suppl. 3), S548–S559 10.1002/mds.22062 18781672

[B29] Qureshi F. Morales A. Elble R. J. (1996). Tremor due to infarction in the ventrolateral thalamus. Mov. Disord. 11, 440–444 10.1002/mds.870110416 8813227

[B30] Reich S. G. (2010). Pearls: hyperkinetic movement disorders. Semin. Neurol. 30, 15–22 10.1055/s-0029-1245005 20127576

[B31] Rostasy K. Augood S. J. Hewett J. W. Leung J. C. Sasaki H. Ozelius L. J. (2003). TorsinA protein and neuropathology in early onset generalized dystonia with GAG deletion. Neurobiol. Dis. 12, 11–24 10.1016/S0969-9961(02)00010-4 12609485

[B32] Rub U. Heinsen H. Brunt E. R. Landwehrmeyer B. den Dunnen W. F. A. Gierga K. (2009). The human premotor oculomotor brainstem system; can it help to understand oculomotor symptoms in Huntington’s disease? Neuropathol. Appl. Neurobiol. 35, 4–15 10.1111/j.1365-2990.2008.00994.x 19187058

[B33] Rub U. Hoche F. Brunt E. R. Heinsen H. Seidel K. Del Turco D. (2012). Degeneration of the cerebellum in Huntington’s disease (HD): possible relevance for the clinical picture and potential gateway to pathological mechanisms of the disease process. Brain Pathol. [Epub ahead of print]. 10.1111/j.1750-3639.2012.00629.x 22925167 PMC8029117

[B34] Saka E. Goodrich C. Harlan P. Madras B. K. Graybiel A. M. (2004). Repetitive behaviors in monkeys are linked to specific striatal activation patterns. J. Neurosci. 24, 7557–7565 10.1523/JNEUROSCI.1072-04.2004 15329403 PMC6729641

[B35] Schneider J. A. Mirra S. S. (1994). Neuropathologic correlates of persistent neurologic deficit in lithium intoxication. Ann. Neurol. 36, 928–931 10.1002/ana.410360621 7998783

[B36] Schuurman P. R. Bosch D. A. Bossuyt P. M. Bonsel G. J. van Someren E. J. de Bie R. M. (2000). A comparison of continuous thalamic stimulation and thalamotomy for suppression of severe tremor. N. Engl. J. Med. 342, 461–468 10.1056/NEJM200002173420703 10675426

[B37] Seidel K. Brunt E. R. de Vos R. A. I. Dijk F. van der Want H. J. Rub U. (2009). The p62 antibody reveals various cytoplasmic protein aggregates in spinocerebellar ataxia type 6. Clin. Neuropathol. 28, 344–349 19788049 10.5414/npp28344

[B38] Seidel K. den Dunnen W. F. A. Schultz C. Paulson H. Frank S. de Vos R. A. I. (2010). Axonal inclusions in spinocerebellar ataxia type 3. Acta Neuropathol. 120, 449–460 10.1007/s00401-010-0717-7 20635090 PMC2923324

[B39] Seidel K. Meister M. Dugbartey G. J. Zijlstra M. P. Vinet J. Brunt E. R. (2011). Cellular protein quality control and the evolution of aggregates in SCA3. Neuropathol. Appl. Neurobiol. 38, 548–558 10.1111/j.1365-2990.2011.01220.x 21916928

[B40] Seidel K. Meister M. Dugbartey G. J. Zijlstra M. P. Vinet J. Brunt E. R. (2011). Cellular protein quality control and the evolution of aggregates in SCA3. Neuropathol. Appl. Neurobiol. 38, 548–558 10.1111/j.1365-2990.2011.01220.x 21916928

[B41] Seidel K. Siswanto S. Brunt E. R. den Dunnen W. F. A. Korf H. W. Rub U. (2012). Brain pathology of spinocerebellar ataxias. Acta Neuropathol. 124, 1–21 10.1007/s00401-012-1000-x 22684686

[B42] Shoulson I. (1986). On chorea. Clin. Neuropharmacol. 9(Suppl. 2), S85–S99 10.1097/00002826-198612030-00009 3297318

[B43] Smith Y. (2012). “Functional anatomy of the basal ganglia” in Movement Disorders, 3rd Edn, Chap. 4, eds Watts R. L. Standaert D. Obeso J. A. (McGraw Hill), 67–88

[B44] Standaert D. G. (2011). Update on the pathology of dystonia. Neurobiol. Dis. 42, 148–151 10.1016/j.nbd.2011.01.012 21220015 PMC3073692

[B45] Vonsattel J. P. Myers R. H. Stevens T. J. Ferrante R. J. Bird E. D. Richardson E. P. Jr. (1985). Neuropathological classification of Huntington’s disease. J. Neuropathol. Exp. Neurol. 44, 559–577 10.1097/00005072-198511000-00003 2932539

[B46] Walker R. H. Brin M. F. Sandu D. Good P. F. Shashidharan P. (2002). TorsinA immunoreactivity in brains of patients with DYT1 and non-DYT1 dystonia. Neurology 58, 120–124 10.1212/WNL.58.1.120 11781416

[B47] Zweig R. M. Hedreen J. C. Jankel W. R. Casanova M. F. Whitehouse P. J. Price D. L. (1988). Pathology in brainstem regions of individuals with primary dystonia. Neurology 38, 702–706 10.1212/WNL.38.5.702 3362365

